# SMRT-mediated co-shuttling enables export of class IIa HDACs independent of their CaM kinase phosphorylation sites

**DOI:** 10.1111/jnc.12058

**Published:** 2012-11-15

**Authors:** Francesc X Soriano, Sangeeta Chawla, Paul Skehel, Giles E Hardingham

**Affiliations:** * Centre for Integrative Physiology, University of EdinburghEdinburgh, UK; † Department of Cell Biology, Faculty of Biology, University of BarcelonaBarcelona, Spain; ‡ Department of Biology, University of YorkYork, UK

**Keywords:** calcium channels, histone deacetylase, neurodegeneration, neurotrophic factor

## Abstract

The Class IIa histone deacetylases (HDAC)4 and HDAC5 play a role in neuronal survival and behavioral adaptation in the CNS. Phosphorylation at 2/3 N-terminal sites promote their nuclear export. We investigated whether non-canonical signaling routes to Class IIa HDAC export exist because of their association with the co-repressor Silencing Mediator Of Retinoic And Thyroid Hormone Receptors (SMRT). We found that, while HDAC5 and HDAC4 mutants lacking their N-terminal phosphorylation sites (HDAC4^MUT^, HDAC5^MUT^) are constitutively nuclear, co-expression with SMRT renders them exportable by signals that trigger SMRT export, such as synaptic activity, HDAC inhibition, and Brain Derived Neurotrophic Factor (BDNF) signaling. We found that SMRT's repression domain 3 (RD3) is critical for co-shuttling of HDAC5^MUT^, consistent with the role for this domain in Class IIa HDAC association. In the context of BDNF signaling, we found that HDAC5^WT^, which was more cytoplasmic than HDAC5^MUT^, accumulated in the nucleus after BDNF treatment. However, co-expression of SMRT blocked BDNF-induced HDAC5^WT^ import in a RD3-dependent manner. In effect, SMRT-mediated HDAC5^WT^ export was opposing the BDNF-induced HDAC5 nuclear accumulation observed in SMRT's absence. Thus, SMRT's presence may render Class IIa HDACs exportable by a wider range of signals than those which simply promote direct phosphorylation.

Histone acetylation and deacetylation plays an important regulatory role in gene transcription, with acetylation strongly associated with increased transcriptional activity [for review (Haberland *et al*. 2009; Perissi *et al*. 2010, 2004)]. Acetylation/deacetylation of histones is a dynamic process that depends on the balance between activity of histone acetyltransferases (HAT) and histone deacetylases (HDAC). The HDACs are a large protein family named after their ability to remove acetyl groups from lysine residues located within the N-terminal tail of histones (Haberland *et al*. 2009; Perissi *et al*. 2010), but can act on non-histones as well. In mammals, there are five classes of HDACs (Class I, IIa, IIb, III, and IV) containing many different HDACs (18 in humans). Class IIa HDACs (HDAC4, 5, 7, 9), unlike other HDACs, are expressed in a tissue-specific manner with high expression levels in skeletal, cardiac, and smooth muscle, bone, the immune system, the vascular system, and the brain [reviewed in (Parra and Verdin 2010)]. Studies with knockout mice for various Class IIa HDACs show their important function in differentiation and developmental processes such as formation of skeletal muscle, cardiac hypertrophy, bone development and T-cell differentiation (Martin *et al*. 2007; Haberland *et al*. 2009; Parra and Verdin 2010). In the CNS, Class IIa HDACs controls neuronal survival (Linseman *et al*. 2003; Bolger and Yao 2005; Chen and Cepko 2009), differentiation (Schneider *et al*. 2008), long-term-memory-related synaptic plasticity (Guan *et al*. 2002), and behavioral responses (Tsankova *et al*. 2006; Renthal *et al*. 2007; Taniguchi *et al*. 2012).

A characteristic of Class IIa HDACs is that their ability to suppress transcription in the nucleus is subject to dynamic regulation. Class IIa HDACs are subject to phosphorylation on two or three conserved serine residues in their N-termini domain, and this modification exposes a nuclear export signal and creates a binding site for 14-3-3 proteins in the cytoplasm which anchor them there, thus preventing them from acting as transcriptional co-repressors (McKinsey *et al*. 2001; Martin *et al*. 2007; Parra and Verdin 2010). Mutation of these phosphorylation sites renders Class IIa HDACs ‘non-exportable’. The primary kinase family responsible for signal-dependent Class IIa phosphorylation and subsequent export are the Ca^2+^/calmodulin-dependent protein kinases (CaM kinases), although other kinases such as PKD and SIK are also capable of acting at these sites. In neurons, the major Class IIa HDACs expressed are HDAC4 and HDAC5, and these are subject to activity- and CaM kinase-dependent nucleo-cytoplasmic shuttling (Chawla *et al*. 2003). Moreover, CaM kinase-dependent nuclear exclusion of HDAC5 has been implicated in promoting the survival of neurons (Linseman *et al*. 2003). HDAC4, in contrast may play pro-survival or pro-death roles in the nucleus which may depend on the neuron's developmental stage (Bolger and Yao 2005; Majdzadeh *et al*. 2008). HDAC4 has been additionally shown to have an active pro-survival role in retinal neurons when resident in the cytoplasm (Chen and Cepko 2009). Furthermore, alterations to Class IIa activity mediate the actions of certain anti-depressants and control addiction pathways (Tsankova *et al*. 2006; Renthal *et al*. 2007; Taniguchi *et al*. 2012). Thus, signaling mechanisms that alter the subcellular localization of Class IIa HDACs have the capacity to influence important aspects of neuronal fate and function.

Class IIa HDACs can exist in the nucleus as part of multi-protein co-repressor complexes centred on SMRT or its close relative N-CoR, and containing other co-repressor proteins and Class I HDACs, particularly HDAC3 (Huang *et al*. 2000; Guenther *et al*. 2001; Perissi *et al*. 2004; Haberland *et al*. 2009; Watson *et al*. 2012). Indeed, one function of Class IIa HDACs is thought to be as a physical bridge between the SMRT/N-CoR complex and certain transcription factors such as myocyte enhancer factor 2 (MEF2), rather than contributing substantial HDAC activity as such (Fischle *et al*. 2002; Lahm *et al*. 2007). Interaction of Class IIa HDACs with SMRT is through SMRT's repression domain 3 (RD3) (Huang *et al*. 2000; Fischle *et al*. 2002). SMRT or N-CoR are known to undergo nuclear export in response to certain stimuli, including EGF-induced MEK1 signaling or Akt activation, and dissociation from chromatin in response to IκB kinase (Hong and Privalsky 2000; Hermanson *et al*. 2002; Hoberg *et al*. 2006; Perissi *et al*. 2004, 2010). In neurons, activity-dependent Ca^2+^ transients trigger SMRT export via a combination of MEK1 and CaM kinase pathways (McKenzie *et al*. 2005; Soriano *et al*. 2011). Other stimuli are also known to promote SMRT export, such as inhibition of Class I HDAC activity, specifically HDAC3 (Soriano and Hardingham 2011).

Thus, while CaM kinase signaling is capable of promoting the nuclear export of both Class IIa HDACs and SMRT, certain signaling pathways are selective for one or the other co-repressor. Given that SMRT and Class IIa HDAC family members associate with each other this raises the possibility, hitherto untested, that SMRT may be able to co-shuttle Class IIa HDACs out of the nucleus independent of the classical phosphorylation site-dependent mechanism. By studying the movement of ‘non-exportable’ phospho-site mutants of HDAC4 and 5, we find that SMRT, acting via its RD3 domain, is able to co-shuttle HDAC4/5 out of the nucleus in response to SMRT-exporting stimuli. Relevance of this pathway is shown in the context of BDNF signaling. We show that BDNF-induced MEK1 signaling promotes HDAC5 import, but that in the presence of SMRT this import is canceled out because of the promotion of SMRT export by MEK1 signaling which acts to co-shuttle HDAC5 back out of the nucleus via SMRT's RD3 domain.

## Methods

### Neuronal cultures and stimulations

Cortical neurons from E21 Sprague Dawley rats were cultured as described (Leveille *et al*. 2010), growth medium was comprised of Neurobasal A medium + B27 (Invitrogen, Paisley, UK), 1% rat serum, 1 mM glutamine. Experiments were performed after a culturing period of 9–10 days during which cortical neurons develop a rich network of processes, express functional NMDA-type and AMPA/kainate-type glutamate receptors, and form synaptic contacts. Stimulations were performed after transferring neurons into defined medium lacking trophic support ‘TMo’ (Papadia *et al*. 2005): 10% minimal essential medium (Invitrogen), 90% Salt-Glucose-Glycine (SGG) medium (SGG: 114 mM NaCl, 0.219% NaHCO_3_, 5.292 mM KCl, 1 mM MgCl_2_, 2 mM CaCl_2_, 10 mM HEPES, 1 mM Glycine, 30 mM Glucose, 0.5 mM sodium pyruvate, 0.1% Phenol Red; osmolarity 325 mosm/L, hereafter TMo). Stimulations were initiated approximately 48 h after transfection. Bursts of action potential firing were induced by treatment of neurons with 50 μM bicuculline, and burst frequency was enhanced by addition of 250 μM 4-amino pyridine (Hardingham *et al*. 2001; Baxter *et al*. 2011). Trichostatin-A (TSA, 1 μM) was added for 8 h, which induces substantial histone H3 and H4 acetylation (Soriano *et al*. 2009b). BDNF (25 ng/mL) was also added for 8 h. extracellular signal-regulated kinase (ERK)1/2 inhibitor PD98059 (50 μM) was added 30 min before the stimulations.

### Plasmids

SMRT-myc and green fluorescent protein (GFP)-SMRTα full length were gifts from Martin Privalsky [UC Davis; (Hong and Privalsky 2000)]. The MEF2 luciferase reporter containing three binding sites for MEF2 transcription factors was a gift from Eric Olson (Lu *et al*. 2000a). Plasmids GFP-SMRT^RD3^ (SMRT^Δ(1018-1523)^), GFP-SMRT^ΔRD4^ (GFP-SMRT^Δ(1523-1854)^), and RD3-myc have been described elsewhere (Soriano and Hardingham 2011). The HDAC5^WT^-Flag-GFP expression vector has been described previously (Belfield *et al*. 2006) and a non-exportable version of it (HDAC5^MUT^-Flag-GFP) was generated by using overlap extension PCR to mutate serines 259 and 498 of HDAC5 to alanine residues. For this study, both HDAC5^WT^-Flag-GFP and HDAC5^MUT^-Flag-GFP vectors were modified for use in this study by introducing a stop codon between the Flag and GFP ORFs by site-directed mutagenesis (primer: TGA TGA TGA TAA ATC TtG Ata GGT GAG CAA GGG CG, underlined nucleotides indicate mutations) to prevent expression of the GFP portion. The resulting vectors are referred to as HDAC5^WT^-Flag and HDAC5^MUT^-Flag. HDAC4^WT^-Flag (a gift from Tony Kouzarides) has been described previously, as has HDAC4^MUT^-Flag containing mutations to its 3 CaM kinase sites S246/467/632A as described previously (Miska *et al*. 2001).

### Transfections and immunofluorescence

Neurons were transfected using Lipofectamine 2000 (Invitrogen) as described (Soriano *et al*. 2006) and stimulated 48 h after transfection. Immunofluorescence was performed as described (Soriano *et al*. 2008). Cells were fixed in 4% paraformaldehyde, 3% sucrose in phosphate-buffered saline for 20 min. Permeabilization was performed with 0.5% NP-40 in phosphate-buffered saline. Primary antibodies used were anti-flag to visualize HDAC4/5 localization (1 : 1000; Sigma, Gillingham, UK), anti-GFP antibody to visualize SMRT localization (1 : 700; Invitrogen). Antibody binding was detected using biotinylated secondary antibody/cy3-conjugated streptavidin, and nuclei were counter-stained with 4′,6-diamidino-2-phenylindole. Non-saturating pictures were taken on a Leica AF6000 LX imaging system (Leica Microsystems, Wetzlar, Germany), with a DFC350 FX digital camera and the subcellular distribution scored. For each treatment, ca. 150–200 cells were analyzed within three to five independent experiments. Distribution was scored either as exclusively nuclear or as containing observable cytoplasmic localization (Al-Mubarak *et al*. 2009).

### Luciferase reporter assay

Firefly luciferase-based MEF2 reporter vector (MRE-Luc) was transfected along with a renilla expression vector (pTK-RL), and also, HDAC5^WT^-Flag or HDAC5^MUT^-Flag. Neurons were stimulated (where appropriate) for 8 h with BDNF (25 ng/mL) 40 h after transfection. Luciferase assays were performed using the Dual Glo assay kit (Promega, Fitchburg, Wisconsin, USA) with Firefly luciferase-based reporter activity normalized to the Renilla control (pTK-RL plasmid) in all cases.

### Statistical analysis

Statistical testing involved a two-tailed paired Student's *t*-test. For any multiple comparisons within data sets we used a one-way anova followed by Fisher's LSD *post-hoc* test.

## Results

### SMRT can mediate nuclear export of “non-exportable” mutants of Class IIa HDACs

Synaptic activity controls the expression of many genes through regulating the activity or expression of DNA-binding transcription factors (West *et al*. 2001; Papadia *et al*. 2008; Soriano *et al*. 2009a). However, activity-dependent signal pathways also influence the activity and location of broad-specificity transcriptional coactivators and co-repressors (Chawla *et al*. 2003; McKenzie *et al*. 2005; Soriano *et al*. 2011). We previously showed that synaptic activity promotes the nuclear export of SMRT in neurons mediated by both CaM kinase signaling and MEK1 signaling (McKenzie *et al*. 2005). As discussed above, SMRT is known to co-localize with Class IIa HDACs HDAC4 and HDAC5 in several cell types including in neurons and this association is important for SMRT's function as a co-repressor. The localization of Class IIa HDACs is subject to dynamic regulation by nuclear CaM kinase activation, which phosphorylates HDAC4 and HDAC5, triggering nuclear export (Haberland *et al*. 2009). However, given the association of HDAC4/5 with SMRT, we hypothesized that SMRT may have the capacity to promote Class IIa HDAC export independent of the canonical direct phosphorylation pathway, in response to signals that trigger SMRT export.

**Fig. 1:**
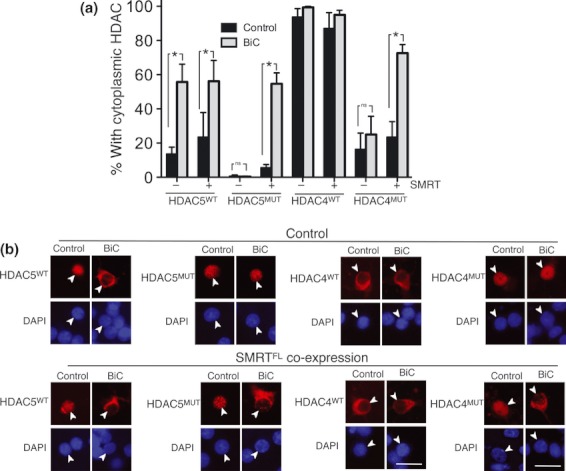
Silencing mediator of retinoic and thyroid hormone receptors (SMRT) mediates non-canonical pathway to Class IIa histone deacetylase (HDAC) nuclear export. (a) Class IIa HDACs lacking CaMKIV phosphorylation sites (HDAC^MUT^) can be exported when co-expressed with SMRT. Neurons were transfected with the indicated HDAC-flag constructs in the presence or absence of full-length green fluorescent protein (GFP)-SMRT, and 48 h after transfection, the neurons were stimulated for 8 h with bicuculline plus 4-AP (labeled BiC in this and subsequent figures) or left unstimulated and the cellular localization of the HDAC-flags was analyzed. *p < 0.05 (*n* = 3–5). (b) Examples of the cellular localization of the indicated flag-tagged HDACs in absence or presence of SMRT after treatment with BiC for 8 h. Scale bar is 20 μm here and thereafter.

To investigate this, we first utilized a mutant of HDAC5 lacking its two CaM kinase phosphorylation sites, rendering it non-exportable via the classical pathway. All HDAC constructs used in this study contained C-terminal flag tag enabling expression to be detected by immunofluorescence with an anti-flag antibody. In agreement with previous studies, in resting neurons, wild-type HDAC5 was predominantly nuclear but also at significant levels in the cytoplasm (Fig. 1a). In contrast, HDAC5^MUT^ was found to be almost exclusively nuclear (Fig. 1a). As expected, HDAC5^MUT^ was not exported from the nucleus in response to strong synaptic activity, while synaptic activity promoted the efficient nuclear export of HDAC5 (Fig. 1a and b), consistent with previous studies (Chawla *et al*. 2003). We next investigated whether co-expression of SMRT could affect the signal-responsiveness of the localization of HDAC5^MUT^. We previously showed that HDAC5 and SMRT co-localize in the nucleus, and that in response to synaptic activity HDAC5 is first exported from the nucleus, followed by SMRT (McKenzie *et al*. 2005). Consistent with this, we found that co-expression of SMRT had no effect on the activity-dependent export of wild-type HDAC5. We also found that co-expression of HDAC5^MUT^ with SMRT had little effect on the basal localization of HDAC5^MUT^ (Fig. 1a and b). Strikingly, however, in the presence of SMRT, HDAC5^MUT^ was exported in response to synaptic activity (Fig. 1a and b). We also looked at the influence of SMRT on the localization of a ‘non-exportable’ mutant of HDAC4 [HDAC4^MUT^, (Grozinger and Schreiber 2000)] containing serine-to-alanine mutations at serines 246/467/632 (Note that wild-type HDAC4 was found to be constitutively cytoplasmic (Fig. 1a and b), in agreement with previous studies (Chawla *et al*. 2003) and so was not investigated further). We found that co-expression of SMRT resulted in HDAC4^MUT^ being exported from the nucleus in response to synaptic activity (Fig. 1a). Thus, the ‘non-exportable’ mutants of HDAC4 and HDAC5 can be rendered exportable by SMRT co-expression, suggestive of a non-canonical pathway to Class IIa HDAC export.

### SMRT's RD3 domain is important for co-shuttling of HDAC5

**Fig. 2:**
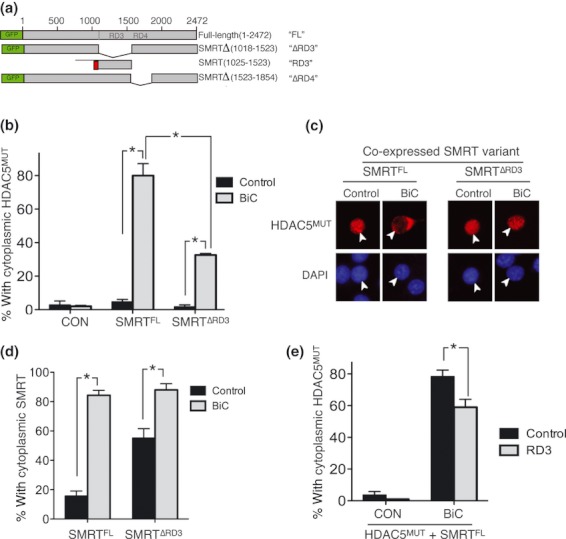
Silencing mediator of retinoic and thyroid hormone receptors (SMRT)'s repression domain 3 (RD3) domain is important for mediating export of histone deacetylase (HDAC)5. (a) Schematic of SMRT deletion mutants/fragments used in this study. The red box indicates a myc tag. (b) Deletion of SMRT RD3 interacting domain impairs SMRT-dependent HDAC5^MUT^ export. HDAC5^MUT^-Flag was co-expressed in neurons together with full-length green fluorescent protein (GFP)-SMRT (SMRT^FL^), SMRT lacking the RD3 domain (GFP-SMRT^Δ^^RD^^3^) or globin as a control plasmid (CON, here and thereafter) and were stimulated for 8 h with BiC or left unstimulated and the cellular localization of the HDAC5^MUT^-Flag was analyzed. *p < 0.05 (*n* = 3). (c) Examples of the cellular localization of HDAC5^MUT^-flag when co-expressed with the indicated GFP-SMRT variants after treatment with BiC for 8 h. (d) GFP-SMRT^FL^ and SMRT^Δ^^RD^^3^ are exported by synaptic activity. Analysis of cellular localization of SMRT^FL^ or GFP-SMRT^Δ^^RD^^3^ after stimulation with BiC for 8 h. *p < 0.05 (*n* = 3). (e) Disruption of HDAC5 interaction with SMRT impairs SMRT-dependent HDAC5^MUT^ export. Neurons were co-transfected with HDAC5^MUT^-flag plus GFP-SMRT^FL^ and the SMRT's RD3-myc domain to disrupt SMRT-HDAC interaction or globin (control) and HDAC5^MUT^-flag cellular localization was analyzed after stimulation with BiC. *p < 0.05 (*n* = 3).

We decided to investigate this putative co-shuttling mechanism further, focussing on HDAC5. Association of SMRT with Class IIa HDACs relies, at least in part on an interaction with SMRT's RD3 domain (Huang *et al*. 2000; Fischle *et al*. 2002). We therefore investigated whether deletion of this domain affected the ability of SMRT to promote activity-dependent export of HDAC5^MUT^. We used a mutant of SMRT we made where amino acids 1018–1523, encompassing the RD3 domain were deleted (Fig. 2a, hereafter SMRT^ΔRD3^). We confirmed our recent findings that SMRT^ΔRD3^ undergoes activity-dependent export as does full-length SMRT (SMRT^FL^) (Soriano *et al*. 2011) (Fig. 2d). However, co-expression of SMRT^ΔRD3^ was far worse at promoting activity-dependent export of HDAC5^MUT^ than SMRT^FL^ (Fig. 2b and c). As SMRT's RD3 domain when expressed on its own is not subject to activity-dependent export (Soriano *et al*. 2011), we reasoned that expression of it, by competing with full-length HDAC5^MUT^ for HDAC5, may interfere with activity-dependent export of HDAC5^MUT^ by SMRT^FL^ and this was indeed observed (Fig. 2e). Collectively, these experiments support a role for the Class IIa HDAC-interacting RD3 domain in SMRT-mediated co-shuttling of Class IIa HDACs.

### HDAC inhibition promotes SMRT-mediated co-shuttling of HDAC5

**Fig. 3:**
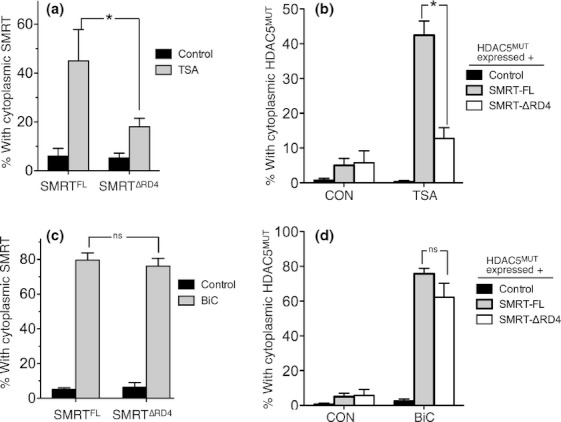
Silencing mediator of retinoic and thyroid hormone receptors (SMRT)-dependent HDAC^MUT^ export requires SMRT export. (a) Trichostatin-A (TSA)-dependent SMRT export relies on SMRT's repression domain 4 (RD4) domain. Neurons were transfected with green fluorescent protein (GFP)-SMRT^FL^ or GFP-SMRT lacking the RD4 domain (SMRT^Δ^^RD^^4^) and stimulated with TSA (1 μM) for 8 h and cellular the localization was analyzed. *p < 0.05 (*n* = 4). (b) Deletion of SMRT RD4 interacting domain abolish TSA induced SMRT-dependent HDAC5^MUT^ export. HDAC5^MUT^-Flag was co-expressed with the indicated SMRT constructs, stimulated for 8 h with TSA and the cellular localization of the flag-HDAC5^MUT^ was analyzed. *p < 0.05 (*n* = 4–5). (c) Synaptic activity-dependent SMRT export does not require the RD4 domain. Neurons were transfected with GFP-SMRT^FL^ or GFP-SMRT^Δ^^RD^^4^ and 48 h later stimulated with BiC for 8 h and cellular the localization was analyzed. *p < 0.05 (*n* = 5). (d) Deletion of SMRT RD4 interacting does not affect the synaptic activity induced SMRT-dependent HDAC5^MUT^ export. HDAC5^MUT^-flag was co-expressed with the indicated SMRT constructs, stimulated for 8 h with BiC and the cellular localization of the HDAC5^MUT^-Flag was analyzed. *p < 0.05 (*n* = 4–5).

We recently showed that inhibition of Class I HDAC (specifically HDAC3) activity promoted SMRT export via a mechanism dependent on its RD4 domain (Soriano and Hardingham 2011), confirmed in Fig. 3a. In that study, we presented data that support a model whereby SMRT's RD4 region can recruit factors capable of mediating nuclear export of SMRT, but whose function and/or recruitment is suppressed by HDAC3 activity (Soriano and Hardingham 2011). As the RD4 domain is not required for *activity*-dependent export of SMRT (Soriano *et al*. 2011), we can exploit this to further illustrate SMRT-mediated export of HDAC5^MUT^. Inhibition of HDAC activity by TSA treatment had little effect on basal HDAC5^MUT^ nuclear localization in isolation (Fig. 3b). However, when SMRT was co-expressed, TSA strongly promoted SMRT export in a manner dependent on the RD4 domain (Fig. 3b). By comparison, activity-dependent SMRT export is independent of the RD4 domain (Soriano and Hardingham 2011), Fig. 3c and consistent with that, SMRT-mediated HDAC5^MUT^ export in response to synaptic activity was independent of the RD4 domain (Fig. 3d).

### BDNF has opposing effects on SMRT and HDAC5 localization

**Fig. 4:**
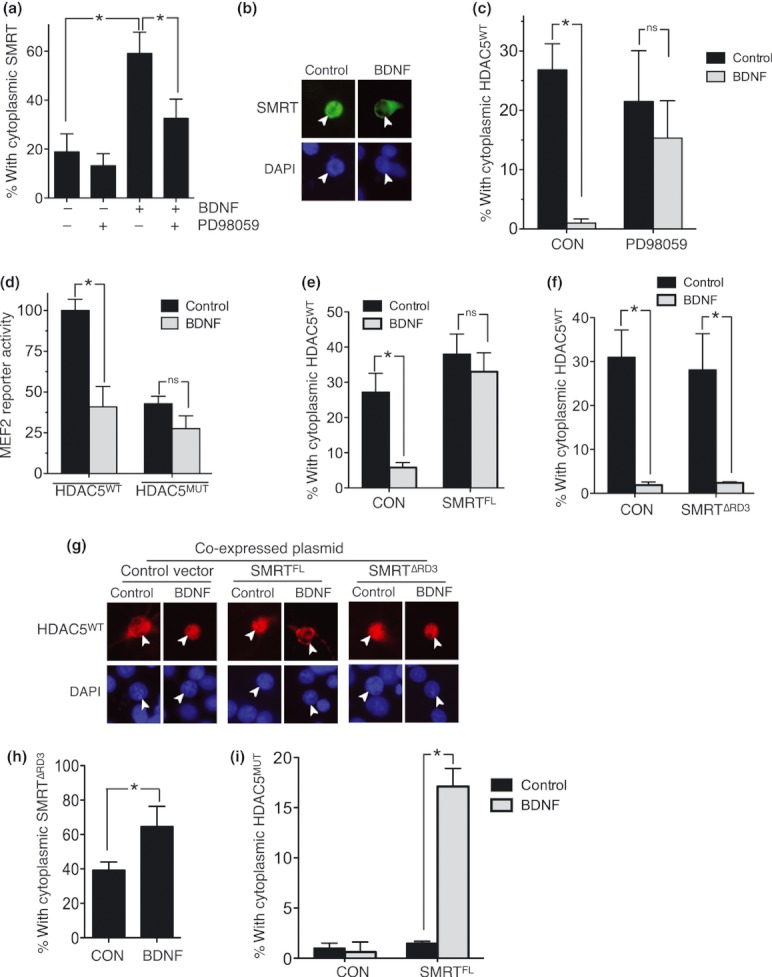
The presence of silencing mediator of retinoic and thyroid hormone receptors (SMRT) determines histone deacetylase (HDAC)5 cellular localization after brain derived neurotrophic factor (BDNF) stimulation. (a) BDNF induces SMRT nuclear export. Neurons were transfected with green fluorescent protein (GFP)-SMRT^FL^ and its cellular localization was analyzed after stimulation with BDNF (25 ng/mL) for 8 h. *p < 0.05 (*n* = 4). (b) Examples of the cellular localization of SMRT after treatment with BDNF. (c) BDNF promotes HDAC5 nuclear localization in an extracellular signal-regulated kinase (ERK)1/2-dependent manner. Neurons were transfected with HDAC5^WT^-flag and its cellular localization was analyzed after stimulation with BDNF (25 ng/mL) for 8 h in absence or presence of the ERK1/2 inhibitor PD98059 (50 μM). *p < 0.05 (*n* = 6). (d) BDNF represses myocyte enhancer factor 2 (MEF2)-mediated gene expression. *p < 0.05 (*n* = 3). Neurons were transfected with a luciferase reporter containing 3 MEF2 binding sites plus either HDAC5^WT^-flag or HDAC5^WT^-flag, then treated with BDNF for 8 h, 40 h post-transfection. *p < 0.05 (*n* = 3). (e) BDNF-dependent HDAC5 nuclear import is blocked by SMRT. Neurons were transfected with HDAC5^WT^-flag in the presence or absence of GFP-SMRT and HDAC5^WT^-flag cellular localization was analyzed after stimulation with BDNF (25 ng/mL) for 8 h. *p < 0.05 (*n* = 5). (f) Inhibition of BDNF mediated HDAC5 import is dependent of SMRT's repression domain 3 (RD3) domain. HDAC5^WT^-flag was co-expressed in neurons together with full-length GFP-SMRT lacking the RD3 domain (SMRT^Δ^^RD^^3^) or globin as a control plasmid (CON) and 48 h after transfection the neurons were stimulated for 8 h with BDNF or left unstimulated and the cellular localization of the HDAC5^WT^-flag was analyzed. *p < 0.05 (*n* = 4). (g) Representative examples of the cellular localization of HDAC5^WT^-flag induced by BDNF in absence or presence of the indicated GFP-SMRT constructs. (h) GFP-SMRT^Δ^^RD^^3^ is exported by BDNF. Analysis of GFP-SMRT^Δ^^RD^^3^ cellular localization after BDNF treatment. *p < 0.05 (*n* = 4). (i) BDNF mediated SMRT export promotes HDAC5^MUT^ export. Neurons were transfected with HDAC5^MUT^-flag in the presence or absence of GFP-SMRT and HDAC5^MUT^-flag cellular localization was analyzed after stimulation with BDNF (25 ng/mL) for 8 h. *p < 0.05 (*n* = 4).

Activity-dependent Ca^2+^ signals are capable of triggering CaM kinase-dependent HDAC5 export independent of any SMRT co-shuttling mechanism (Chawla *et al*. 2003). We therefore wanted to know what type of signal the co-shuttling mechanism could be relevant for, that is, a signal that triggers SMRT export but not direct nuclear Ca^2+^/CaM kinase-dependent HDAC5 export. We decided to study signaling by the neurotrophin BDNF, as activation of the BDNF receptor TrkB strongly activates ERK1/2 signaling, but evokes modest Ca^2+^ mobilization compared with action potential bursting. We found that exposure of neurons to BDNF caused SMRT export in a substantial proportion of neurons (Fig. 4a and b), and that this was reversed when ERK1/2 activation was blocked by pre-incubation with the MEK1 inhibitor PD98059 (Fig. 4a). We next studied the influence of BDNF signaling on wild-type HDAC5 localization expressed in the absence of SMRT. Not only did we find that BDNF signaling failed to promote HDAC5 export, it actually triggered increased nuclear localization of HDAC5 in a ERK1/2-dependent manner (Fig. 4c).

These observations indicate that BDNF treatment could affect gene expression mediated by HDAC5-repressed transcription factors such as MEF2s (McKinsey *et al*. 2001). To investigate this, we co-expressed a MEF2-reporter (Lu *et al*. 2000a) with either HDAC5^WT^ or HDAC5^MUT^ and assayed reporter activity in the presence or absence of BDNF treatment. BDNF treatment repressed MEF2 reporter activity in the presence of HDAC5^WT^ (Fig. 4d), consistent with it increasing nuclear localization of HDAC5. Indeed, the effect of BDNF is to repress MEF2 reporter activity to levels observed when the constitutively nuclear HDAC5^MUT^ is expressed. Moreover, BDNF has no additional effect in the presence of HDAC5^MUT^, further suggesting that BDNF is exerting its effects on MEF2-mediated transcription in the presence of HDAC5^WT^ by promoting HDAC5 nuclear import (Fig. 4d).

Given that BDNF signaling promotes HDAC5 nuclear accumulation but SMRT export, and HDAC5 and SMRT associate with each other, we wanted to determine whether the presence of SMRT has any effect on the movement of HDAC5 following BDNF treatment. We found that when SMRT was co-expressed with HDAC5, BDNF-induced nuclear import of HDAC5 was completely blocked (Fig. 4e and g). Moreover, this inhibition of HDAC5 import by SMRT was dependent on SMRT's HDAC5-interaction domain (RD3): expression of SMRT^ΔRD3^ had no effect on BDNF-induced HDAC5 import (Fig. 4f and g), despite still being subjected to BDNF-induced export (Fig. 4h). Collectively, these observations suggest that the direct effect of BDNF on the nuclear accumulation of HDAC5 is being canceled out by the indirect, SMRT-mediated export. Analysis of HDAC5^MUT^ localization confirmed this: BDNF had no effect on the nuclear localization of HDAC5^MUT^ on its own (Fig. 4i). However, in the presence of SMRT, BDNF treatment resulted in the export of HDAC5^MUT^ in a significant number of neurons (Fig. 4i). Thus, the presence of SMRT directly influences the signal-dependent localization of Class IIa HDACs.

## Discussion

Subcellular localization of Class IIa HDACs represents the major mechanism to regulate their function. Phosphorylation at two or three conserved Serine residues at the N-terminus leads to interaction with 14-3-3, nuclear export and derepression of the target genes. Here, we have shown that in neurons Class IIa HDACs can be exported from the nucleus in an independent manner to the classical phosphorylation mechanism, by co-shuttling with SMRT. As several signals are known to be able to influence the subcellular localization of SMRT and its close relative N-CoR, an implication of this work is that the presence of SMRT/N-CoR may render Class IIa HDACs responsive to those signals, particularly given that Class IIa HDACs can exist in SMRT/N-CoR-containing complexes in the nucleus. We have shown that BDNF-induced MEK1 signaling promotes SMRT export but has the reverse effect on HDAC5, promoting import. However, when SMRT is present BDNF-induced HDAC5 import is blocked – likely because of direct import being canceled out by SMRT-mediated export. The influence of SMRT is lost upon deletion of its HDAC4/5-interacting RD3 domain, consistent with a direct co-shuttling mechanism. Other signals may also impact on Class IIa HDAC localization in the presence of SMRT/N-CoR, such as the PI3-kinase/Akt pathway, which in response to CNTF treatment mediates the nuclear export of N-CoR in neural stem cells (Hermanson *et al*. 2002).

Although this is the first time that Class IIa HDAC export has been demonstrated totally independent of their signal-responsive phosphorylation sites, co-shuttling of one class member HDAC5, by another (HDAC4) has been shown (Backs *et al*. 2008). Unlike CaM kinases IV and I, which can promote export of both HDAC4 and 5, CaM kinase II can only promote HDAC4 export because HDAC5 lacks a CaM kinase II docking site (Backs *et al*. 2006). However, in the presence of HDAC4, HDAC5 can be co-shuttled out of the nucleus by CaM kinase II by interacting with HDAC4. Even ‘non-exportable’ HDAC5^MUT^ can be co-shuttled in this way, suggestive of ‘piggy-pack’ export. However, a certain degree of export is also observed if ‘non-exportable’ HDAC4^MUT^ is paired with wild-type HDAC5, indicating that docking of CaM kinase II on HDAC4 followed by trans-phosphorylation of HDAC5 can also lead to export (Backs *et al*. 2006). Importantly, when HDAC5^MUT^ and HDAC4^MUT^ are co-expressed there is no export in response to CaM kinase II activity: phosphorylation sites on either HDAC4 or HDAC5 are essential for the export to take place, unlike the situation we describe where export of HDAC5^MUT^ or HDAC4^MUT^ can be promoted by SMRT. Of note, Class IIa HDACs can themselves co-shuttle other proteins in response to CaM kinase signaling, through direct interaction. For example, both HDAC4 and HDAC5 physically associate with ankyrin-repeat proteins ANKRA2 and RFXANK. Phosphorylation of HDAC by CaM kinases promotes the nuclear export of the HDAC/ANKRA2 and HDAC/RFXANK complexes but not if the HDAC used is a non-exportable mutant (McKinsey *et al*. 2006).

To conclude, the prominent role that Class IIa HDACs play in controlling neuronal survival and death, and differentiation, as well as certain behavioral responses, such as addiction, make an understanding of signals that control their subcellular localization to be of direct importance. Outside of the CNS, the roles played by Class IIa HDACs in regulating transcriptional programs relating to skeletal myogenesis, cardiac hypertrophy, and thymocyte development (Lu *et al*. 2000b; McKinsey *et al*. 2000; Zhang *et al*. 2002; Haberland *et al*. 2009; Parra and Verdin 2010; Watson *et al*. 2012) raise the question as to whether these functions may be influenced by signals that promote SMRT-mediated co-shuttling independent of the classical export pathways.

## Abbreviations

4-AP: 4-amino pyridine
BDNF: brain derived neurotrophic factor
CaM kinases: Ca^2+^/calmodulin-dependent protein kinases
GFP: green fluorescent protein
HAT: histone acetyltransferase
HDAC: histone deacetylase
RD3: repression domain 3
SMRT: silencing mediator of retinoic and thyroid hormone receptors
